# Association between diet soft drink consumption and metabolic dysfunction-associated steatotic liver disease: findings from the NHANES

**DOI:** 10.1186/s12889-023-17223-0

**Published:** 2023-11-20

**Authors:** Yanrui Wu, Zongbiao Tan, Junhai Zhen, Chuan Liu, Jixiang Zhang, Fei Liao, Weiguo Dong

**Affiliations:** 1 Department of Gastroenterology, Renmin Hospital of Wuhan University, Wuhan, China; 2 Department of General Practice, Renmin Hospital of Wuhan University, Wuhan, China

**Keywords:** Diet soft drink, Metabolic dysfunction-associated steatotic liver disease, Body mass index, NHANES, Mediation

## Abstract

**Background:**

Lifestyle change plays a crucial role in the prevention and treatment of metabolic dysfunction-associated steatotic liver disease (MASLD). In recent years, diet soft drinks that emphasize “zero sugar and zero calories” have become all the rage, but whether diet soft drink consumption is associated with MASLD is not clear.

**Methods:**

This study included data from the National Health and Nutrition Examination Surveys (NHANES) in 2003–2006. The assessment of MASLD status primarily relied on the Fatty Liver Index (FLI). Weighted multiple Logistic regression models were constructed to evaluate the association between diet soft drink consumption and MASLD. Additionally, mediation analysis was performed to examine the mediating effect of body mass index (BMI).

**Results:**

A total of 2,378 participants were included in the study, among which 1,089 individuals had MASLD, and the weighted prevalence rate was 43.64%. After adjusting for variables related to demographic, lifestyle, and metabolic syndrome, excessive diet soft drink consumption (the “always” frequency) remained significantly associated with the occurrence of MASLD (*OR* = 1.98, 95%*CI* = 1.36–2.89, *P* = 0.003). It was estimated that 84.7% of the total association between diet soft drink consumption and MASLD was mediated by BMI (*P* < 0.001).

**Conclusions:**

Excessive diet soft drink consumption was associated with the occurrence of MASLD. BMI may play a mediating role in the association between diet soft drink consumption and MASLD.

## Introduction

Metabolic dysfunction-associated steatotic liver disease (MASLD), previously known as non-alcoholic fatty liver disease (NAFLD), was officially renamed in June 2023 [1]. The prevalence of MASLD is increasing at an alarming rate, accounting for 32.4% of the total population in 2022 [2], and it is currently one of the most common chronic liver diseases. MASLD is characterized by steatosis of hepatocytes and excludes the influence of viruses, alcohol, and autoimmune factors [3]. Approximately 20% of patients with MASLD will progress to metabolic dysfunction-associated steatohepatitis (MASH), which is the presence of hepatocellular injury (hepatocyte ballooning), and 20% of patients with MASH will develop cirrhosis within 30 to 40 years [4]. However, no drug has been approved for the treatment of MASLD at present, and fat content in the body can only be reduced by controlling diet and increasing exercise, thereby alleviating or reversing steatosis of the liver [5, 6]. Therefore, lifestyle change plays a crucial role in the prevention and treatment of MASLD. It is interesting and meaningful to find out the dietary patterns that may increase the risk of MASLD.

Soft drink is a general term for a class of water-based non-alcoholic beverages, which has no standard definition and usually contains natural or artificial sweeteners. Common carbonated drinks are classified as soft drinks, while coffee, tea, milk, cocoa, and undiluted fruit and vegetable juices are not considered soft drinks [7]. Given the high calorie content of sugar-sweetened beverages, an association with MASLD has long attracted attention [8]. In addition, fructose, one of the natural sweeteners commonly found in soft drinks, has been shown to induce insulin resistance and inflammation in the liver, thus promoting the development of MASLD [9]. In recent years, diet soft drinks that emphasize “zero sugar and zero calories” have become all the rage due to consumers’ concerns about health issues, but they usually contain artificial sweeteners such as aspartame. In fact, much attention has been paid to the effects of diet soft drinks or artificial sweeteners on the body. Several cross-sectional and cohort studies have shown that diet soft drink consumption is associated with increased body mass index (BMI) and percentage body fat in adolescents [10–12]. In other words, diet soft drinks that emphasize zero calories may not necessarily prevent weight gain, on the contrary, excessive intake may also lead to obesity. Whereas obesity will bring a series of metabolic problems, so diet soft drink consumption may also increase the risk of metabolic syndrome. Analysis of data from the Northern Manhattan Study suggested that daily diet soft drink consumption was associated with an increased risk of vascular events [13]. In addition, previous studies have also shown that diet soft drink consumption is associated with increased blood pressure in adolescents [14], and excessive intake of artificial sweeteners increases the risk of type 2 diabetes [15]. However, the association between diet soft drinks and MASLD is not clear. We hypothesized that excessive diet soft drink consumption mediates the development of MASLD through an increase in BMI.

Therefore, this study intends to explore the association between diet soft drink consumption and MASLD using data obtained from the National Health and Nutrition Examination Surveys (NHANES), and to evaluate the mediating effect of BMI in this association by mediation analysis.

## Methods

### Study population

**Fig. 1:**
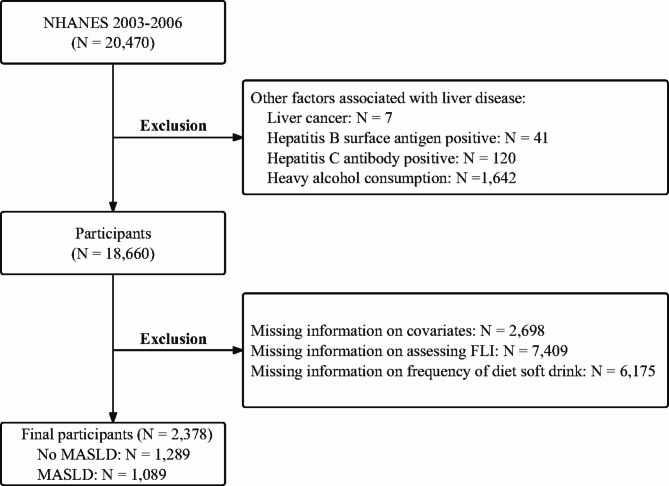
Flowchart of sample selection from the NHANES 2003–2006

NHANES is an important survey program sponsored by the National Center for Health Statistics (NCHS) in the United States (www.cdc.gov/nchs/nhanes.htm), aiming to assess the health and nutritional status of the American population. Since detailed records of participants’ diet soft drink intake were only available during the 2003–2006 dietary interviews, this study primarily included data from these two cycles for analysis (accessed on 2 July 2023). The following criteria were used for exclusion: (1) Liver diseases that may be associated with other factors (such as positive hepatitis B surface antigen or positive hepatitis C antibody, severe alcohol consumption); (2) Missing information of diet soft drink consumption frequency data; (3) Missing information of data for assessing MASLD; (4) Missing information of data on smoking, physical activity, hypertension, dyslipidemia, diabetes, and other conditions. The specific selection process is shown in Fig. 1.

### Assessment of MASLD

The initial stage of MASLD is characterized by abnormal accumulation of fat in liver tissue. Since the NHANES database from 2003 to 2006 lacked data on liver transient elastography, the assessment of MASLD status primarily relied on the Fatty Liver Index (FLI) [16]. The specific calculation formula for FLI was as follows:

$$\begin{aligned}&FLI=\frac{{e}^{0.953\times \text{ln}\left(triglycerides\right)+0.139\times BMI+0.718\times \text{ln}\left(gamma-glutamyl\,transferase\right)+0.053\times waist\,circumference-15.745}}{1+{e}^{0.953\times \text{ln}\left(triglycerides\right)+0.139\times BMI+0.718\times \text{ln}\left(gamma-glutamyl\,transferase\right)+0.053\times waist\,circumference-15.745}}\quad\times 100\end{aligned}$$

In the exclusion of other liver diseases associated with the aforementioned factors, when FLI ≥ 60, we considered the participant to have MASLD.

### Evaluation of diet soft drink consumption

The assessment of diet soft drink consumption frequency was based on participants’ responses to the question “How often do you drink diet soft drinks?” in the food frequency questionnaire. We redefined the participants’ diet soft drink consumption frequency as follows: “Almost never or never” was defined as “Never,” “About 1/4 of the time” was defined as “Rarely,” “About 1/2 of the time” was defined as “Sometimes,” and “About 3/4 of the time” and “Almost always” were defined as “Always.”

### Covariates

To determine the association between diet soft drink consumption and MASLD, we adjusted for the following potential confounding factors: age, gender (male, female), race/ethnicity (non-Hispanic white, non-Hispanic black, Mexican American, and other), educational level (less than high school, high school, and more than high school), smoking status (never, former, and now) [17], average daily physical activity level (no, mild, moderate, and heavy), carbohydrate intake, dietary fiber intake, polyunsaturated fatty acid (PUFA) intake, hypertension (yes, no), hyperlipidemia (yes, no), and diabetes mellitus (yes, no).

### Statistical analysis

**Fig. 2:**
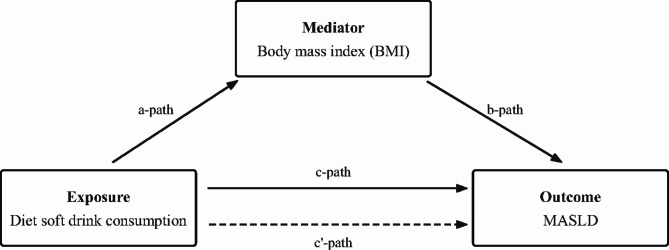
Path diagram of the mediation analysis models

Since NHANES adopted complex multistage probability sampling, we followed the guidelines of NCHS to select “wtmec2yr” as weight variable in the analysis process. Continuous variables were described by mean ± standard deviation (SD), and *P* value was calculated by weighted linear regression model. Categorical variables were described by weighted percentages, and *P* value was calculated by weighted chi-square test. Weighted multiple Logistic regression models were constructed to evaluate the relationship between diet soft drink consumption and MASLD, and the results were expressed as odds ratio (*OR*) and 95% confidence interval (*CI*). To examine the mediating effect of BMI in the association of diet soft drink consumption with MASLD, we carried out a mediation analysis using the “mediation” package of R [18]. As shown in Fig. 2, the mediation models included three pathways: a) exposure to mediator; b) mediator to outcome; c) exposure to outcome (total effect) [19]. And total effect was the sum of direct effect (c’) and mediated (indirect) effect. The proportion of the mediated effect was calculated as (mediated effect/total effect) × 100%.

All statistical analyses were performed using R (version 4.3.0). Statistical significance was defined as *P* < 0.05.

## Results

### Characteristics of participants

Among 2,378 people who met our selection criteria and had all available information, 1,089 had MASLD, and the weighted prevalence rate was 43.64% (22,837,939/52,328,574). Participant characteristics after weighting are shown in Table 1. The mean age and BMI of MASLD group were 52.17 ± 15.23 years and 33.92 ± 6.15 kg/m^2^, while the mean age and BMI of non-MASLD group were 49.06 ± 17.27 years and 24.70 ± 3.28 kg/m^2^. There were significant differences in age and BMI distribution between the two groups (both *P* < 0.001). Males accounted for 54.60% of the MASLD population, more than females (*P* < 0.001). In terms of diet soft drink intake frequency, MASLD group was significantly higher than non-MASLD group (*P* < 0.001), especially the proportion of “always” (38.65% vs. 28.68%). However, there were no statistical differences in the intake of energy, carbohydrate, dietary fiber, and PUFA between the two groups (all *P* > 0.05). In addition, there were significant differences in the distribution of race, education level, physical intensity, and smoking status between the two groups (all *P* < 0.05). And it is worth noting that the prevalence of hypertension (53.39% vs. 30.60%), hyperlipidemia (88.42% vs. 67.16%), and diabetes mellitus (22.61% vs. 6.08%) in MASLD population was significantly higher than that in non-MASLD population (all *P* < 0.001).

**Table 1:** Survey-weighted characteristics of participants classified by liver status

Variable	No MASLD (*N* = 29,490,635)	MASLD (*N* = 22,837,939)	*P* value
Age, *Mean* ± *SD* (years)	49.06 ± 17.27	52.17 ± 15.23	< 0.001
BMI, *Mean* ± *SD* (kg/m^2^)	24.70 ± 3.28	33.92 ± 6.15	< 0.001
FLI, *Mean* ± *SD*	26.03 ± 17.25	83.73 ± 12.01	< 0.001
Energy intake, *Mean* ± *SD* (kcal/d)	2,137.69 ± 935.77	2,194.37 ± 971.01	0.223
Carbohydrate intake, *Mean* ± *SD* (g/d)	261.73 ± 119.81	262.11 ± 127.77	0.955
Dietary fiber intake, *Mean* ± *SD* (g/d)	16.57 ± 9.20	16.03 ± 8.99	0.180
PUFA intake, *Mean* ± *SD* (g/d)	17.86 ± 11.17	17.92 ± 11.51	0.914
**Gender**, ***n*(%)**			
Male	11,254,415 (38.16)	12,470,130 (54.60)	< 0.001
Female	18,236,220 (61.84)	10,367,809 (45.40)	
**Race**, ***n*(%)**			
Black	2,616,194 (8.87)	2,628,231 (11.51)	0.014
White	22,464,048 (76.17)	17,365,814 (76.04)	
Mexican	1,699,085 (5.76)	1,485,682 (6.51)	
Other	2,711,308 (9.19)	1,358,212 (5.95)	
**Education level**, ***n*(%)**			
Less than high school	1,552,942 (5.27)	1,466,644 (6.42)	0.002
High school	9,624,893 (32.64)	9,145,431 (40.04)	
More than high school	18,312,800 (62.10)	12,225,864 (53.53)	
**Diet soft drink consumption**, ***n*(%)**			
Never	15,722,533 (53.31)	9,546,596 (41.80)	< 0.001
Rarely	1,488,279 (5.05)	1,313,197 (5.75)	
Sometimes	3,821,605 (12.96)	3,151,320 (13.80)	
Always	8,458,218 (28.68)	8,826,826 (38.65)	
**Physical intensity**, ***n*(%)**			
No	5,694,783 (19.31)	3,460,523 (15.15)	< 0.001
Mild	5,801,320 (19.67)	6,538,080 (28.63)	
Moderate	16,006,289 (54.28)	11,597,612 (50.78)	
Heavy	1,988,243 (6.74)	1,241,723 (5.44)	
**Smoke**, ***n*(%)**			
Never	16,745,750 (56.78)	11,094,447 (48.58)	0.004
Former	7,626,610 (25.86)	7,423,224 (32.50)	
Now	5,118,275 (17.36)	4,320,268 (18.92)	
**Hypertension**, ***n*(%)**			
No	20,466,525 (69.40)	10,645,657 (46.61)	< 0.001
Yes	9,024,110 (30.60)	12,192,282 (53.39)	
**Hyperlipidemia**, ***n*(%)**			
No	9,685,989 (32.84)	2,644,232 (11.58)	< 0.001
Yes	19,804,646 (67.16)	20,193,707 (88.42)	
**Diabetes mellitus**, ***n*(%)**			
No	27,696,909 (93.92)	17,675,152 (77.39)	< 0.001
Yes	1,793,726 (6.08)	5,162,787 (22.61)	

MASLD: metabolic dysfunction-associated steatotic liver disease; BMI: body mass index; FLI: fatty liver index; PUFA: polyunsaturated fatty acid

### Association of diet soft drink consumption with MASLD

To assess the association between diet soft drink consumption and the occurrence of MASLD, we constructed three weighted multiple Logistic regression models and the results are presented in Table 2. Model 1 mainly adjusted demographic variables such as age, gender, race, and education level, model 2 adjusted lifestyle variables (smoking status, physical activity intensity, carbohydrate intake, dietary fiber intake, and PUFA intake) on the basis of model 1, and model 3 adjusted variables related to metabolic syndrome (hypertension, hyperlipidemia, and diabetes mellitus) on the basis of model 2. Obviously, when taking the “never” frequency as the reference, the “always” frequency was significantly associated with the occurrence of MASLD in all three models (model 1: *OR* = 2.04, 95%*CI* = 1.54–2.70, *P* < 0.001; model 2: *OR* = 2.12, 95%*CI* = 1.56–2.88, *P* < 0.001; model 3: *OR* = 1.98, 95%*CI* = 1.36–2.89, *P* = 0.003).

**Table 2:** Association between diet soft drink consumption and MASLD

Diet soft drink consumption	Model 1		Model 2		Model 3	
	*OR* (95% *CI*)	*P* value	*OR* (95% *CI*)	*P* value	*OR* (95% *CI*)	*P* value
Never (ref)						
Rarely	1.61 (0.93, 2.77)	0.084	1.61 (0.91, 2.86)	0.096	1.60 (0.87, 2.94)	0.116
Sometimes	1.41 (1.02, 1.94)	**0.038**	1.41 (1.00, 1.97)	0.050	1.35 (0.91, 2.03)	0.123
Always	2.04 (1.54, 2.70)	**< 0.001**	2.12 (1.56, 2.88)	**< 0.001**	1.98 (1.36, 2.89)	**0.003**

Model 1: Adjusted for age, gender, race, and education level

Model 2: Additional adjustment for smoking, physical intensity, carbohydrate intake, dietary fiber intake, and PUFA intake on the basis of Model 1

Model 3: Additional adjustment for hypertension, hyperlipidemia, and diabetes mellitus on the basis of Model 2

ref = reference. *P* value in bold indicates statistical significance

### Subgroup analysis

In order to observe the association between diet soft drink consumption and MASLD in different gender, smoking status, and metabolic conditions, we carried out subgroup analyses of the above factors. As shown in Table 3, the association between the frequency of diet soft drink consumption and the occurrence of MASLD was not significant in the diabetic population (*P* for trend = 0.547), whereas the association seemed to be consistent under other stratification factors (all *P* for trend < 0.05). Moreover, an interaction was observed between diet soft drink consumption and diabetes mellitus for the occurrence of MASLD (*P* for interaction = 0.033).

**Table 3:** Association between diet soft drink consumption and MASLD by different group stratification

Character	Never (ref)	Rarely	*P*	Sometimes	*P*	Always	*P*	*P* for trend	*P* for interaction
**Gender**									
Male		1.11 (0.56, 2.18)	0.757	1.49 (0.93, 2.39)	0.090	2.55 (1.78, 3.67)	< 0.001	< 0.001	0.052
Female		2.33 (1.04, 5.22)	0.041	1.26 (0.84, 1.87)	0.243	1.98 (1.36, 2.89)	0.001	0.002	
**Smoke**									
Never		1.77 (0.83, 3.77)	0.130	1.44 (0.95, 2.20)	0.085	2.05 (1.32, 3.20)	0.003	0.003	0.639
Former		1.34 (0.47, 3.81)	0.566	0.91 (0.54, 1.52)	0.697	1.74 (1.06, 2.85)	0.030	0.041	
Now		1.91 (0.65, 5.64)	0.221	2.36 (1.08, 5.15)	0.033	2.07 (1.03, 4.18)	0.043	0.018	
**Hypertension**									
No		2.39 (1.30, 4.40)	0.008	1.68 (1.06, 2.64)	0.029	2.15 (1.35, 3.43)	0.003	0.002	0.065
Yes		0.75 (0.35, 1.62)	0.439	1.16 (0.77, 1.74)	0.462	1.81 (1.24, 2.63)	0.004	0.003	
**Hyperlipidemia**									
No		1.74 (0.57, 5.29)	0.306	0.89 (0.30, 2.60)	0.813	2.75 (1.38, 5.49)	0.007	0.012	0.213
Yes		1.61 (0.92, 2.81)	0.091	1.36 (0.95, 1.94)	0.087	1.84 (1.34, 2.52)	< 0.001	< 0.001	
**Diabetes mellitus**									
No		1.71 (1.01, 2.89)	0.046	1.43 (0.99, 2.05)	0.054	1.94 (1.34, 2.82)	0.002	0.001	0.033
Yes		0.49 (0.10, 2.39)	0.351	0.53 (0.23, 1.27)	0.144	0.81 (0.41, 1.59)	0.508	0.547	

Adjusted for age, race, education level, physical intensity, carbohydrate intake, dietary fiber intake, and PUFA intake

### Mediation analysis

In order to verify the aforementioned hypothesis, we analyzed the relationship between diet soft drink consumption and MASLD using BMI as a mediating variable. The mediation analysis was performed based on adjustment for age, gender, race, education level, smoking status, physical activity intensity, carbohydrate intake, dietary fiber intake, PUFA intake, hypertension, hyperlipidemia, and diabetes mellitus. As presented in Table 4, diet soft drink consumption was positively associated with BMI (*P* < 0.001) and positively associated with MASLD (*P* < 0.001). In addition, BMI was positively associated with the occurrence of MASLD (*P* < 0.001). Further, the direct effect of diet soft drink consumption on MASLD was not statistically significant (*P* = 0.230), but the mediated effect was significant (*P* < 0.001). It was estimated that 84.7% of the total association between diet soft drink consumption and MASLD was mediated by BMI (*P* < 0.001).

**Table 4:** Mediation effect of BMI on the association between diet soft drink consumption and MASLD

Mediator		Exposure to mediator	Mediator to outcome	Direct effect	Mediated (indirect effect)	Total effect (exposure to outcome)		Proportion mediated (%)
BMI	Estimate	1.558	0.673	0.015	0.085	0.100	84.7	
	*SE*/*CI* 95%	0.266	0.030	(-0.010, 0.040)	(0.056, 0.110)	(0.062, 0.140)		
	*P* value	< 0.001	< 0.001	0.230	< 0.001	< 0.001	< 0.001	

MASLD: metabolic dysfunction-associated steatotic liver disease; BMI: body mass index; Exposure: diet soft drink consumption; Outcome: MASLD; model adjusted for age, gender, race, education level, smoking, physical intensity, carbohydrate intake, dietary fiber intake, PUFA intake, hypertension, hyperlipidemia, and diabetes mellitus

## Discussion

In this large cross-sectional study, we explored the association between diet soft drink consumption and MASLD using data obtained from the NHANES.

The weighted prevalence rate of MASLD was 43.64% in the present study, which was slightly higher than that estimated by epidemiological studies, because we excluded some individuals with missing key information during the process of inclusion, and FLI was not the gold standard for the diagnosis of MASLD. Of note, although BMI of the MASLD population was significantly higher than that of the non-MASLD population, there was no statistical difference in energy intake between the two groups. The traditional view is that the increase of energy (calorie) intake and the decrease of physical activity lead to obesity, and then a series of metabolic syndrome. However, this concept seems unable to explain the above results. It may be that participants adopted a series of dietary strategies to control energy intake after diagnosis of MASLD or realization of excessive BMI, or they underreported energy intake on the questionnaire. In Logistic regression analyses, excessive diet soft drink consumption (the “always” frequency) remained significantly associated with MASLD after adjusting for variables related to demographic, lifestyle, and metabolic syndrome. And mediation analysis demonstrated that a large proportion (84.7%) of the association between diet soft drink consumption and MASLD was mediated by BMI. However, whether diet soft drinks cause weight gain is still inconclusive. One theory is that the artificial sweeteners in diet soft drinks stimulate appetite and cause sugar cravings, thereby increasing the consumption of other sugary or calorie-dense foods and leading to weight gain [20]. In the subgroup analysis defined by diabetes, no association was observed between diet soft drink consumption and MASLD in diabetic population, and similar results were obtained in a previous study from Tianjin, China [21], but the specific reasons are currently unclear and warrant further exploration.

The term soft drink originated to distinguish flavored drinks from hard liquor or distilled spirits and was recommended as a substitute to change the heavy drinking habits of early Americans [7], while the rise of diet soft drinks was related to concerns about obesity. But in recent years, the health problems caused by diet soft drinks have gradually attracted attention. A growing number of studies have shown that diet soft drink consumption is associated with an increased risk of obesity, type 2 diabetes, and other features of metabolic syndrome [10, 14, 15]. The current view is that the health risks of diet soft drinks mainly come from artificial sweeteners, such as aspartame and sucralose. As recently as July 2023, the International Agency for Research on Cancer (IARC) of the World Health Organization (WHO) classified aspartame as possibly carcinogenic to humans (IARC Group 2B). As for the mechanism by which artificial sweeteners increase the risk of MASLD, it should be multifaceted. In addition to BMI as a mediator concerned in this study, previous animal studies have shown that artificial sweeteners can promote insulin resistance [22], and induce glucose intolerance by altering intestinal microbiota [23], both of which are closely related to the pathogenesis of MASLD.

To the best of our knowledge, this is the first study to explore the association between diet soft drink consumption and MASLD in a nationally representative sample, and our findings can provide valuable dietary recommendations for the prevention and treatment of MASLD. Of course, this study also has several limitations. First of all, as a cross-sectional study, the impact of reverse causality cannot be excluded, that is, those participants diagnosed with MASLD may be more inclined to choose diet soft drinks. Secondly, FLI was used for the diagnosis of MASLD in this study, rather than the imaging method commonly used in clinical practice, although FLI has been validated and used in a number of previous studies [24]. Finally, the current study was not able to adjust for genetic variants and sleep patterns, which were also risk factors for MASLD [25, 26], due to the lack of genetic information in the NHANES database and large differences in sleep questionnaire content between the two cycles used in this study. In the future, prospective randomized controlled trials are necessary to provide stronger evidence for our findings.

## Conclusions

By analyzing nationally representative data, we found that excessive diet soft drink consumption was associated with the occurrence of MASLD. Additionally, BMI may play a mediating role in the association between diet soft drink consumption and MASLD. Our findings can provide valuable dietary recommendations for the prevention and treatment of MASLD.

## Data Availability

The datasets for this study can be found in the NHANES database (https://www.cdc.gov/nchs/nhanes/index.htm).
